# GABAergic Neuron Activation in the RMTg‐VTA Pathway Modulates Dopaminergic Neuron Excitability and Social Stress Susceptibility in Male Mice

**DOI:** 10.1002/cns.70855

**Published:** 2026-04-01

**Authors:** Guang‐Yue Ma, Jin‐Zhu Zhuang, Shu‐Feng Li, Yu‐E Zhang, Di Zhang, Jun Zhao, Zhen Peng, Liu Yang, Zhang Cao, Xin Xie, Rong Jiang, Hui Sun

**Affiliations:** ^1^ Department of Pathology Binzhou Medical University Hospital Binzhou Shandong Province China; ^2^ Department of Physiology Binzhou Medical University Yantai Shandong Province China; ^3^ Department of Neurobiology, School of Basic Medical Sciences Shandong University Jinan Shandong Province China; ^4^ School of Life Sciences and Health University of Health and Rehabilitation Sciences Qingdao Shandong Province China; ^5^ Yantai Affiliated Hospital of Binzhou Medical University Yantai Shandong Province China; ^6^ Institute of Neuroscience Binzhou Medical University Yantai Shandong Province China; ^7^ Department of Pharmacology Binzhou Medical University Yantai Shandong Province China

**Keywords:** γ‐aminobutyric acid A receptors, dopaminergic neurons, rostral medial tegmental nucleus, social stress, ventral tegmental area

## Abstract

**Background:**

Chronic social stress is a significant risk factor for neuropsychiatric disorders such as anxiety and depression, with the ventral tegmental area (VTA) playing a pivotal role. Although chronic social defeat stress (CSDS) induces adaptations in VTA dopaminergic (DA) neurons, the role of GABAergic modulation in regulating DA neuron excitability and related behaviors remains unclear.

**Methods:**

In this study, we investigated the effects of GABA_A_ receptor activation on VTA DA neuron activity and social stress behaviors in a CSDS model. We employed electrophysiological recordings, immunohistochemistry, and optogenetic manipulations to assess changes in DA neuron excitability and GABAergic input from the rostromedial tegmental nucleus (RMTg).

**Results:**

Our results show that susceptible mice exhibit enhanced spontaneous firing of VTA DA neurons and increased GABA_A_ receptor expression. Pharmacological activation of GABA_A_ receptors suppressed DA neuron hyperactivity and reversed social avoidance behavior. Moreover, optogenetic stimulation of RMTg GABAergic projections to the VTA significantly reduced DA neuron firing and mitigated social avoidance in susceptible mice.

**Conclusion:**

These findings suggest that activation of RMTg GABAergic neurons projecting to VTA effectively reduces DA neuron excitability in the VTA and plays a critical role in the modulation of stress‐induced behavioral deficits, offering a potential therapeutic target for neuropsychiatric disorders.

## Graphical abstract

In susceptible mice, dopamine neurons in the VTA exhibit increased excitability along with upregulation of GABA_A_ receptors. Administration of the GABA_A_ receptor agonist muscimol reduces this neuronal hyperexcitability and reverses social avoidance behavior.

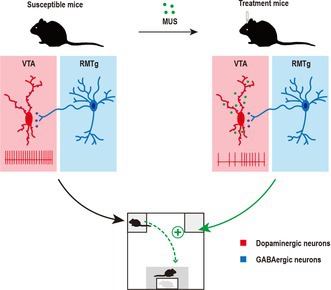

## 1 Introduction

Social stress plays a pivotal role in facilitating adaptation to physical and psychological threats, thereby achieving homeostasis [1, 2, 3]. Chronic social stress exposure is linked to various neuropsychiatric disorders, including pathological anxiety, post‐traumatic stress disorder, and major depressive disorder [4]. The ventral tegmental area (VTA) is a crucial component of the brain's reward and motivation systems, undergoing abnormal adaptations of dopaminergic (DA) neurons in response to chronic social defeat stress (CSDS), which contribute to social stress behavior [5]. Dopamine released from VTA DA neurons, closely associated with motivation and reward processing, is significantly modulated by stress [6]. Furthermore, the mesolimbic system, originating from DA neurons in the VTA, projects to the nucleus accumbens (NAc) and plays a crucial role in regulating motivational behaviors related to reward and aversion [7, 8, 9].

Within the VTA, GABAergic neurons form synapses and release GABA from axon terminals, which binds to GABA receptors and regulates the excitability of DA neurons [10, 11]. The excitatory regulation of DA neurons by GABA neurons via GABA_A_ receptors remains unexplored in the context of social stress behaviors in CSDS mice. Furthermore, DA neurons in the VTA receive inhibitory GABAergic inputs from various sources, including local VTA interneurons, the rostral medial tegmental nucleus (RMTg) [12], and the NAc [13]. Previous research showed that VTA DA neurons received direct monosynaptic GABA inputs from the NAc, and the NAc‐VTA circuit can reverse anxiety‐like behavior induced by chronic emotional stress, but not social stress behavior [14]. GABAergic neurons in the RMTg project to VTA DA neurons, mediating the rewarding effects of cocaine, and may serve as a potential target for therapeutic intervention [15]. However, the precise mechanisms by which these GABAergic circuits influence social stress behaviors remain unclear.

In this study, we aim to investigate the effect of GABAergic neurons in the RMTg projecting to the VTA on the function of DA neurons in the VTA and their involvement in modulating social stress behaviors induced by CSDS. By combining optogenetic manipulation, pharmacological interventions, and electrophysiological recordings, we examine how GABAergic neurons in the RMTg regulate the excitability of VTA DA neurons and how this regulation impacts social defeat‐induced behavioral phenotypes. Our findings provide novel insights into the neural circuitry underlying chronic stress adaptation and offer potential new targets for the development of antidepressant therapies.

## 2 Methods

### 2.1 Animals

Male C57BL/6J mice (8–12 weeks old) were obtained from Jinan Pengyue Experimental Animal Breeding Co. Ltd. (China), while C57BL/6‐Gt (ROSA)26Sor ^tm1(CAG‐GFP, −Mars*L274G) Esm^/J (#028071) and DAT‐Ires‐Cre ^(B6.SJL‐Slc6a3 tm1.1(cre)Bkmn)^/J (#006660) mice were purchased from Jackson Laboratory and were crossed to label DA neurons in the VTA region fluorescently. The male wild type and gene‐crossed mice were housed under standard laboratory conditions, including a 12‐h light/dark cycle, a temperature range of 22°C–25°C, and 40%–60% relative humidity, with ad libitum access to food and water. All mice were maintained in a specific pathogen‐free (SPF) facility. Animal care and experimental procedures were conducted in accordance with institutional and international ethical guidelines and were approved by the University's Animal Welfare Committee, Animal Ethics number: 2021–054.

### 2.2 CSDS Model Preparation

The CSDS model was established following a previously described protocol [16]. CD1 aggressor mice were pre‐screened based on attack latency (≤ 60 s) and attack duration (> 3 min), with selection confirmed over two consecutive days. Male C57BL/6J and gene‐crossed mice were subjected to daily 10‐min encounters with novel CD1 aggressors for 10 consecutive days. Following each session, mice were housed in the same cage as their aggressor but separated by a perforated Plexiglas divider for 24 h. Each experimental mouse faced a different CD1 aggressor each day.

### 2.3 Social Interaction Test

Social interaction was measured in a two‐stage social interaction test. In the first stage (target absence), experimental mice were placed in an open field (42 cm x 42 cm x 42 cm) with an empty screen shell (10 cm wide x 6.5 cm deep x 42 cm high) on the wall of the area [17]. To perform the social interaction test, C57BL/6J mice were placed in an interaction test box and allowed to move freely for 2.5 min in the absence of an unfamiliar caged CD1 mouse. Mice were then returned to the home cage for 30 s. In the second phase, experimental mice were reintroduced into the arena, and a new aggressive CD1 mouse was placed in a barbed wire shell. The time spent in the interaction and corner regions of C57BL/6J mice was recorded and analyzed to identify subpopulations of susceptible and resilient mice. In the presence of CD1 mice, if the experimental mice spend significantly less time in the interaction zone than in the absence of CD1 mice, then these experimental mice would qualify as susceptible mice with social stress behavior. The mice that responded to CD1 mice but did not have reduced the interaction time were termed resilient mice. These mice were randomly divided into groups, which were single blinded to the drug treatment at the time of the test.

### 2.4 Open Field Test

During the experiment, each mouse was gently placed in the center of an open‐field arena (42 cm × 42 cm × 42 cm) with minimal external interference. After placement, the animal was allowed to freely explore the arena for 1 min before formal behavioral recording began; the formal behavioral recording was then conducted for 5 min [14]. At the end of the trial, the mouse was gently returned to its home cage. Prior to testing the next subject, any feces were removed with a tissue and the arena was thoroughly wiped with 75% ethanol to maintain consistent experimental conditions across trials. Recorded parameters included the total distance traveled, movement trajectory, and time spent in the central zone of the arena. Behavioral data were analyzed using Smart software, and statistical comparisons of these parameters were performed between experimental groups to evaluate locomotor activity.

### 2.5 Brain Slice Preparation

Electrophysiological recordings were performed on coronal brain slices containing the VTA, prepared as previously described [18]. Mice (8–12 weeks old) were deeply anesthetized with sodium pentobarbital (80 mg/kg, i.p.) and transcardially perfused with ice‐cold, oxygenated (95% O_₂_/5% CO_₂_) artificial cerebrospinal fluid (ACSF) containing (in mM): 2.5 KCl, 1.25 NaH_₂_PO_4_, 25 NaHCO_₃_, 0.5 CaCl_₂_, 7 MgCl_2_, 10 D‐glucose, and 220 sucrose (osmolarity: 295–305 mOsm). The brain was rapidly removed and immersed in ice‐cold cutting solution (same composition as ACSF). Coronal slices (250 μm) were sectioned using a vibratome (VT‐1000S, Leica, Germany) and transferred to a recovery chamber containing normal ACSF (in mM: 129 NaCl, 3 KCl, 1.3 MgCl_2_, 20 NaHCO_₃_, 1.2 KH_2_PO_₄_, 2.4 CaCl_₂_, 3 HEPES, 10 D‐glucose; oxygenated with 95% O_₂_/5% CO_₂_) at 24°C–25°C for at least 1.5 h before recordings.

### 2.6 In Vitro Electrophysiological Recordings

Patch‐clamp recordings were performed using a MultiClamp 700B amplifier (Molecular Devices, USA) under infrared‐differential interference contrast (IR‐DIC) microscopy (BX71, Olympus, Japan). Data were acquired and analyzed using pClamp 10.2 software (Molecular Devices, USA). Recording pipettes (4–6 MΩ) were pulled using a P‐97 micropipette puller (Sutter Instruments) and filled with internal solution (in mM: 129 NaCl, 3 KCl, 1.3 MgCl_₂_, 20 NaHCO_₃_, 1.2 KH_₂_PO_₄_, 2.4 CaCl_₂_, 3 HEPES, 10 glucose; pH 7.35, 285 mOsm). VTA DA neurons in brain slice projecting to NAc were identified by red retrobeads and GFP fluorescent labeling from genetic hybrid mice; the co‐labeling cells were recorded by patch clamp. Spontaneous firing of VTA DA neurons was recorded in the cell‐attached current‐clamp mode. The electrophysiological characteristics of in vitro brain slice DA neurons in the VTA were based on firing rate (< 10 Hz), action potential width (> 1.1 ms from onset to trough), and long‐duration spikes (> 2.0 ms from onset to resting membrane potential) [18]. For miniature inhibitory postsynaptic current (mIPSCs) recordings, whole‐cell voltage‐clamp recordings were performed at a holding potential of +30 mV. Recording pipettes (3–5 MΩ) were filled with an internal solution containing (in mM): 125 potassium gluconate, 20 KCl, 10 HEPES, 2 MgCl_₂_, 4 ATP, and 1 EGTA (pH 7.2–7.4). To isolate GABA_A_ receptor‐mediated mIPSCs, the extracellular solution was supplemented with DNQX (10 μM), APV (1 μM), and TTX (1 μM) [17]. For miniature light‐evoked IPSCs (eIPSCs) recordings, whole‐cell voltage‐clamp recordings were performed at a holding potential of +30 mV. Recording pipettes (3–5 MΩ) were filled with an internal solution containing (in mM): 125 potassium gluconate, 20 KCl, 10 HEPES, 2 MgCl_₂_, 4 ATP, and 1 EGTA (pH 7.2–7.4). To isolate GABA_A_ receptor‐mediated eIPSCs, the extracellular solution was supplemented with DNQX (10 μM), APV (1 μM), and TTX (1 μM). ChR2 was activated with collimated light from an optogenetic light source device (473 nm, 50 Hz, 5 ms pulse duration) [19, 20]. In all experiments, the series resistance was controlled at < 30 MΩ. Recordings were discarded if series resistance exceeded 30 MΩ or fluctuated > 20%.

### 2.7 In Vivo Electrophysiological Recordings

Single‐unit recordings were performed as described previously [18]. Briefly, mice were anesthetized with sodium pentobarbital (80 mg/kg, i.p.), and a glass electrode was inserted into the VTA (coordinates: anterior–posterior, −3.20 mm; medial‐lateral, 0.7 mm; dorsal‐ventral, −4.00 mm from bregma). DA neurons were identified based on standard physiological criteria: triphasic action potential morphology, long‐duration spikes (> 2.0 ms), wide action potential width (> 1.1 ms from onset to trough), slow firing rate (< 10 Hz), and irregular single spiking with occasional bursting. Bursts were defined as two consecutive spikes within 80 ms, terminated by an interspike interval > 160 ms [21]. Signals were amplified (10 Hz–4 kHz) using an Axoclamp 900A preamplifier, digitized with a Digidata 1440A AD converter (Molecular Devices, USA), and analyzed using pClamp 10.2 software.

### 2.8 Brain Stereotaxic Injection

The mice were anesthetized with pentobarbital sodium (80 mg/kg, i.p.). The hair was removed from the back of the head with a clipper, eye ointment was applied to the eyes, and the mice were placed in a stereotaxic framework (RWD, CHN) for intracranial injection. Coordinates were: VTA (Single side: anterior–posterior, −3.20 mm; lateral‐medial, −0.50 mm; dorsal‐ventral, −4.50 mm relative to bregma), NAc medial shell (NAcMed, both sides: anterior–posterior, +1.50 mm; lateral‐medial, ±0.55 mm; dorsal‐ventral, −4.70 mm relative to bregma), NAc lateral shell (NAcLat, both sides: anterior–posterior, +0.98 mm; lateral‐medial, ±1.80 mm; dorsal‐ventral, −4.92 mm relative to bregma), and RMTg (Single side: anterior–posterior, −3.85 mm; lateral‐medial, −0.50 mm; dorsal‐ventral, −4.50 mm relative to bregma) [22, 23]. Red fluorescent retrobeads (200 nL; LumaFluor) were injected on both sides of NAc using a 1 mL Hamilton syringe [24]. AAV9‐GAD65‐Cre (200 nL, serotype titer 5.11*10E 13 vg/ml, WZ Biosciences Inc) was injected into RMTg, and AAV‐Retro‐EF1a‐DIO‐mCherry (200 nL, serotype titer 1.89*10E 13 vg/ml, WZ Biosciences Inc) or AAV‐Retro‐EF1a‐DIO‐hChR2‐EYFP (200 nL, serotype titer 1*10E 13 vg/ml, WZ Biosciences Inc) was injected into VTA [25]. The injection was performed using a 10 mL microinjector (Hamilton) at a rate of 100 nL/min. After the virus injected into mice was fully expressed (at least 3 weeks), the next experiment was carried out.

### 2.9 Cannula Implantation for Direct Injection of Drugs Into the VTA

Mice were anesthetized by i.p. injection of 4% chloral hydrate (400 mg·kg^−1^, i.p) and placed into a stereotaxic apparatus. Mice were bilaterally implanted with a stainless 26‐gauge cannula fitted with obturators (Plastics One) targeting directly above the VTA with the following coordinates: anterior–posterior, −3.20 mm; lateral‐medial, 0.50 mm; dorsal‐ventral, −4.00 mm relative to bregma. The cannulas were secured with dental cement. Then the mice were singly housed, which were allowed to recover for 2 days. After the surgery, mice were housed at 28°C for 8 h to maintain their body temperature. All instruments were autoclaved. The skin of the animals was sterilized with alcohol and iodine to prevent infection after surgery. Mice were placed in a cage with clean sawdust bedding and provided free access to fresh water and food [26].

### 2.10 Optogenetics Method

Three weeks after the virus was expressed, an optical fiber was implanted unilaterally into the VTA of mice at the following coordinates: anterior–posterior, −3.20 mm; medial‐lateral, −0.50 mm; dorsal‐ventral, −4.50 mm relative to bregma. A 473 nm blue laser diode (Crystal Laser, BCL‐473‐050‐M) was connected to an FC/PC adapter via an optical fiber (Thor Labs, BFL37‐200). Blue light pulses were generated using an optogenetic light source device (Newton Technologies; Aurora Series Photogenetic Light Source System). For in vivo experiments, we employed an optogenetic stimulation protocol consisting of blue light (473 nm, 5 mW per side, 50 Hz, 5 ms pulse duration) for ChR2 activation, based on previously established protocols [19, 27]. A similar stimulation paradigm was also applied in in vitro electrophysiological recordings.

### 2.11 Western Blotting

VTA tissues from susceptible and wild type mice were homogenized in TEVP buffer (10 mM Tris‐base, 5 mM NaF, 1 mM Na_₃_VO_₄_, 1 mM EDTA, and 1 mM EGTA). Protein samples (30 μg) were separated by sodium dodecyl sulfate–polyacrylamide gel electrophoresis (SDS‐PAGE) using 12% gels for 1.5 h. Proteins were then transferred onto a polyvinylidene fluoride (PVDF) membrane in a methanol‐containing transfer buffer at 200 mA for 2 h. The membrane was blocked with 5% non‐fat milk (Sigma, USA) for 1 h at room temperature, followed by overnight incubation at 4°C with primary antibodies. The following primary antibodies were used: anti‐GABA_A_ receptor (mouse, 1:1000, Abcam, ab242001) and anti‐β‐tubulin (rabbit, 1:1000, Cell Signaling Technology, #2128). After three 10‐min washes with TBST, the membrane was incubated for 2 h with secondary antibodies: goat anti‐rabbit (1:4000, ORIGENE, ZB‐2301) and goat anti‐mouse (1:4000, ORIGENE, ZB‐2305). Protein signals were detected using a chemiluminescence system (Clinx Science Instruments, Shanghai, China). Densitometric analysis was performed using ImageJ software, and protein levels were quantified as the ratio of the target protein band intensity to that of the β‐tubulin band.

### 2.12 Histology and Imaging

Mice were deeply anesthetized with pentobarbital sodium (80 mg/kg, i.p.) and intracardially perfused with phosphate‐buffered saline (PBS), followed by 4% paraformaldehyde (PFA). Brains were extracted and post‐fixed in 4% PFA at 4°C overnight, then cryoprotected by sequential immersion in 10%, 20%, and 30% sucrose solutions in PBS until they sank. Coronal sections (15 μm thick) encompassing the regions of interest were prepared using a freezing microtome. Sections were washed three times with PBS (5 min each), blocked with 10% fetal bovine serum (FBS; Viva Cell, C04001, China) in PBS for 2 h at room temperature, and incubated overnight at 4°C with primary antibodies: anti‐tyrosine hydroxylase (TH; rabbit, 1:200, EMD Millipore Corp, AB152) and anti‐GABA_A_ receptor (mouse, 1:200, Abcam, ab242001). After primary incubation, sections were rinsed four times with PBS (15 min each) and incubated for 2 h at room temperature with secondary antibodies: FITC‐conjugated goat anti‐rabbit IgG (1:200, Invitrogen, USA) and Alexa Fluor 647‐conjugated goat anti‐mouse IgG (1:200, Invitrogen, USA). Following four additional PBS washes (10 min each), sections were mounted with Fluoromount aqueous mounting medium (Sigma‐Aldrich, USA) and coverslipped.

### 2.13 Statistics and Reproducibility

Statistical analysis was performed using SPSS software (version 23.0). Normality of continuous variables was assessed by the Shapiro–Wilk test. For data conforming to a normal distribution, group comparisons were conducted using the independent samples *t*‐test, one‐way analysis of variance (ANOVA), or paired samples *t*‐test, as appropriate. Continuous variables that did not follow a normal distribution were compared using the Mann–Whitney *U* test (for two independent groups), the Kruskal–Wallis *H* test (for multiple independent groups), or the Wilcoxon signed‐rank test (for paired samples). Categorical variables are expressed as frequency (n) and percentage (%), and group differences were analyzed with the chi‐square test. *P*‐values < 0.05 were interpreted as statistically significant. All data are presented as mean ± SEM.

## 3 Results

### 3.1 Activation of GABA_A_ Receptors on VTA DA Neurons Reversed Social Stress Behavior in Susceptible Mice

**FIGURE 1:**
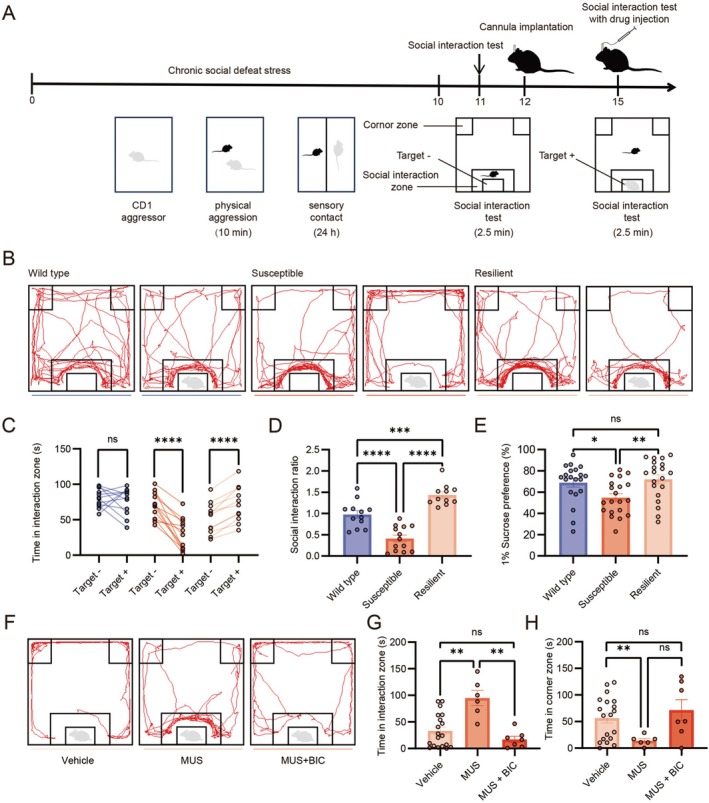
Activation of GABA_A_ receptors reversed social stress behavior in susceptible mice. (A) Timeline for the establishment of the CSDS model, social interaction (SI) test, cannula implantation, and drug injection. (B) Representative traces of wild type, susceptible and resilient mice during the SI test. (C) Time spent in the social interaction zone of wild type (blue), susceptible (red) and resilient (light red) mice (Paired sample *t* test, respectively, wild type: *T* = 0.60, *p* = 0.56, *n* = 12 mice. Susceptible: *T* = 6.78, *p* = 0.00002, *n* = 13 mice. Resilient: T = −8.047, *p* = 0.000011, *n* = 11 mice). (D) Social interaction ratio of wild type (blue), susceptible (red) and resilient (light red) mice (One‐way ANOVA; wild type vs. susceptible: *P* = 0.000024; susceptible vs. resilient: *P* < 0.0001; wild type vs. resilient: *P* = 0.0005; wild type: *N* = 12; susceptible: *N* = 13; resilient: *N* = 11). (E) 1% Sucrose preference of wild type (blue), susceptible (red) and resilient (light red) mice (Kruskal‐wallis test; wild type vs. susceptible: *P* = 0.017; susceptible vs. resilient: *P* = 0.003; wild type vs. resilient: *P* = 0.529; wild type: *N* = 20; susceptible: *N* = 20; resilient: *N* = 20). (F) Representative traces of susceptible mice during the social interaction test treated with vehicle, MUS or/and BIC. (G) Time spent in the social interaction zone of susceptible mice treated with vehicle, MUS or/and BIC (Kruskal‐wallis test; Vehicle vs. MUS: *P* = 0.005; MUS vs. MUS + BIC: *P* = 0.002; Vehicle vs. MUS + BIC: *P* = 0.374; Vehicle: *N* = 19; MUS: *N* = 6; MUS + BIC: *N* = 7). (H) Time spent in the corner zone by susceptible mice treated with vehicle, MUS or/and BIC (One‐way ANOVA; Vehicle vs. MUS: *P* = 0.0011; MUS vs. MUS + BIC: *P* = 0.074; Vehicle vs. MUS + BIC: *P* = 0.88; Vehicle: *N* = 19; MUS: *N* = 6; MUS + BIC: *N* = 7). Data are expressed as mean ± SEM; *p < 0.05, **p < 0.01, ***p < 0.001, ****p < 0.0001, ns means no significant.

Following 10 consecutive days of CSDS, mice underwent the social interaction test on day 11 (Figure 1A). Representative tracking plots showed that, in the presence of an aggressive CD1 mouse, susceptible mice exhibited reduced locomotor activity within the social interaction zone, whereas resilient mice displayed increased exploration of this area (Figure 1B). Consistent with these representative traces, quantitative analysis showed that susceptible mice exhibited a clear social avoidance phenotype, as evidenced by a significant reduction in the time spent in the interaction zone in the presence of a CD1 mouse compared with that in its absence. In contrast, resilient mice spent more time interacting with the social target and displayed a significantly higher interaction ratio (Figure 1C,D). Moreover, susceptible mice exhibited a lower 1% sucrose preference compared to both wild type and resilient mice (Figure 1E). Given the critical role of VTA DA neurons projecting to the NAc in regulating depression‐like behaviors in CSDS mice, in vitro and in vivo firing recordings were performed (Figure S1A), and these DA neurons were identified by the colocalization of red retrobeads and GFP fluorescence in genetically crossed mice (Figure S1B). Representative in vitro and in vivo firing traces are presented (Supplementary Figure S1C,D), and DA neurons were further identified based on their electrophysiological properties during recording [18]. Consistent with previous studies [6, 28], the spontaneous firing frequency of VTA DA neurons projecting to the NAc in susceptible mice brain slices was higher than that observed in wild type mice, as measured by cell‐attached recording (Figure S1E). However, although burst firing is functionally significant, it was absent in VTA slice preparations [21]. The firing frequency of VTA DA neurons and the percentage of spikes in burst firing in susceptible mice in vivo were significantly increased, while no such effects were observed in wild type mice (Figure S1F,G). We further assessed resilient mice and found that the spontaneous firing frequency of VTA DA neurons in resilient mice was significantly lower than that of susceptible mice and did not differ significantly from wild type mice (Figure S1E). Similarly, in vivo recordings revealed that resilient mice exhibited both firing frequency of VTA DA neurons and the percentage of spikes in burst firing that were markedly reduced compared to susceptible mice, while remaining comparable to those in wild type mice (Figure S1F,G).

Previous studies have indicated that DA neurons in the VTA receive axonal projections from both local and external GABAergic neurons, which modulate their function. Given the pivotal role of VTA DA neuron excitability in regulating depression‐like behaviors in CSDS mice, we administered a GABA_A_ receptor agonist (muscimol, MUS) and antagonist (bicuculline, BIC) into the VTA to assess their effects on the behavioral phenotypes of susceptible mice. Initially, we injected vehicle, muscimol, muscimol plus bicuculline into the VTA of susceptible mice, with the “aggressor” CD1 mice (target) present (Figure 1F). Following 10 min of muscimol treatment, susceptible mice showed a significant increase in social interaction time and a concurrent decrease in corner zone time compared to vehicle‐treated mice (Figure 1G,H). In contrast, treatment with both muscimol and bicuculline significantly reduced social interaction time and increased time spent in the corner zone compared to muscimol‐treated mice (Figure 1G,H). These behavioral results indicate that modulation of GABA_A_ receptors in the VTA significantly influences social stress behaviors in susceptible mice after CSDS.

**FIGURE 2:**
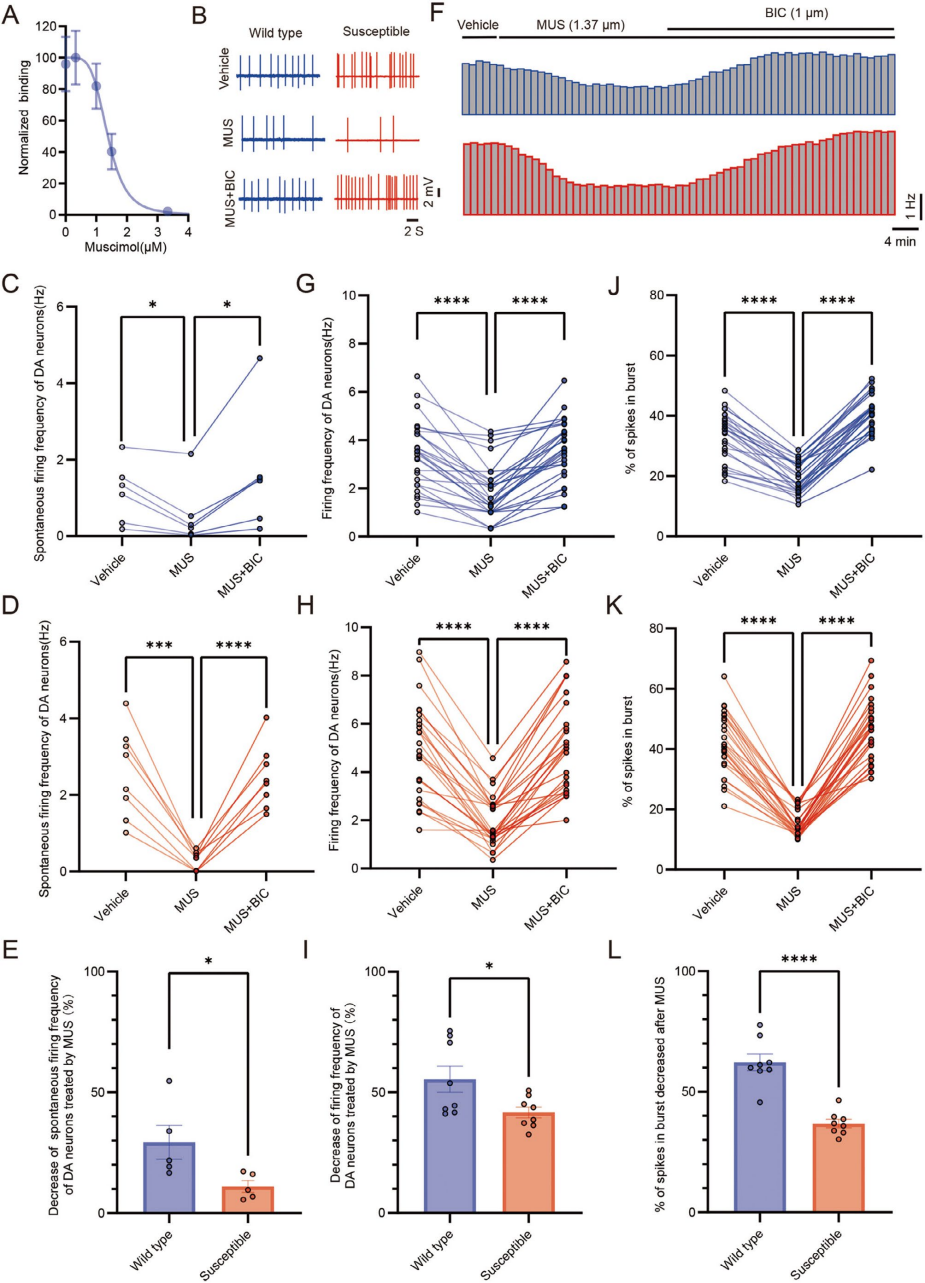
The firing of VTA DA neurons decreased in susceptible mice treated with GABA_A_ agonist. (A) Concentration–response curve for the effect of muscimol decreased spontaneous firing of VTA DA neurons from brain slices fitted with a logistic function in vitro (*n* = 15 cells from 8 wild type mice, IC50 = 1.379 ± 0.6 μM). (B) Typical recordings and action potential waveforms from wild type (blue) and susceptible (red) mice in vitro. (C) and (D) Spontaneous firing frequency of VTA DA neurons in wild type (*n* = 6 cells from 5 wild type mice, Wilcoxon's symbolic rank test: Vehicle vs. MUS: *P* = 0.028, MUS vs. MUS + BIC: *P* = 0.028) and susceptible mice (*n* = 8 cells from 5 wild type mice, Paired sample *t* test: Vehicle vs. MUS: *T* = 6.606, *p* = 0.0003, MUS vs. MUS + BIC: *T* = −8.244, *p* < 0.0001) treated with MUS or/and BIC. (E) Decrease of firing frequency of VTA DA neurons treated by MUS from wild type (blue) and susceptible (red) mice (Two‐tailed, Unpaired *t* test, Susceptible vs. Wild type: *P* = 0.039, *t* = 2.461; wild type: *N* = 5; susceptible: *N* = 5). (F) The time course of the firing frequency of VTA DA neurons with MUS or/and BIC from wild type (blue) and susceptible (red) mice in vivo. (G) and (H) the firing frequency of VTA DA neurons in wild type (*n* = 27 cells from 8 wild type mice, Wilcoxon's symbolic rank test: Vehicle vs. MUS: *P* < 0.0001, MUS vs. MUS + BIC: *P* < 0.0001) and susceptible mice (*n* = 26 cells from 8 susceptible mice, Wilcoxon's symbolic rank test: Vehicle vs. MUS: *P* < 0.0001, MUS vs. MUS + BIC: *P* < 0.0001) injected with MUS or/and BIC. (I) Decrease of firing frequency of VTA DA neurons treated by MUS from wild type (blue) and susceptible (red) mice (Two‐tailed, Unpaired *t* test, Susceptible vs. Wild type: *P* = 0.042, *t* = 2.358; wild type: *N* = 8; susceptible: *N* = 8). (J) and (K) Summary of the effects of MUS or/and BIC on the incidence of burst firing (percentage of spikes in bursts) from wild type (*n* = 27 cells from 8 mice, Paired sample *t* test: Vehicle vs. MUS: *T* = 12.774, *p* < 0.0001, MUS vs. MUS + BIC: *T* = −24.255, *p* < 0.0001) and susceptible mice (*n* = 26 cells from 8 mice, Wilcoxon's symbolic rank test: Vehicle vs. MUS: *P* < 0.0001, MUS vs. MUS + BIC: *P* < 0.0001). (L) Decrease of firing frequency of VTA DA neurons treated by MUS from wild type (blue) and susceptible (red) mice (Two‐tailed, Unpaired *t* test, Susceptible vs. Wild type: *P* < 0.0001, *t* = 6.57; wild type: *N* = 8; susceptible: *N* = 8). Data are expressed as mean ± SEM; *p < 0.05, **p < 0.01, ***p < 0.001, ****p < 0.0001, ns means no significant.

In addition, we also injected vehicle, bicuculline, bicuculline plus muscimol into the VTA of susceptible mice, with the CD1 mice present (Figure 2A). After 10 min of bicuculline treatment, susceptible mice showed no significant differences in social interaction and corner zone time compared to vehicle‐treated mice (Figure S2B,C). Similarly, the combined treatment with muscimol and bicuculline did not yield any significant differences in these measures compared to vehicle‐treated and bicuculline‐treated mice (Figure S2B,C).

### 3.2 The Firing of VTA DA Neurons Decreased in Susceptible Mice Treated With GABA_A_ Agonist

**FIGURE 3:**
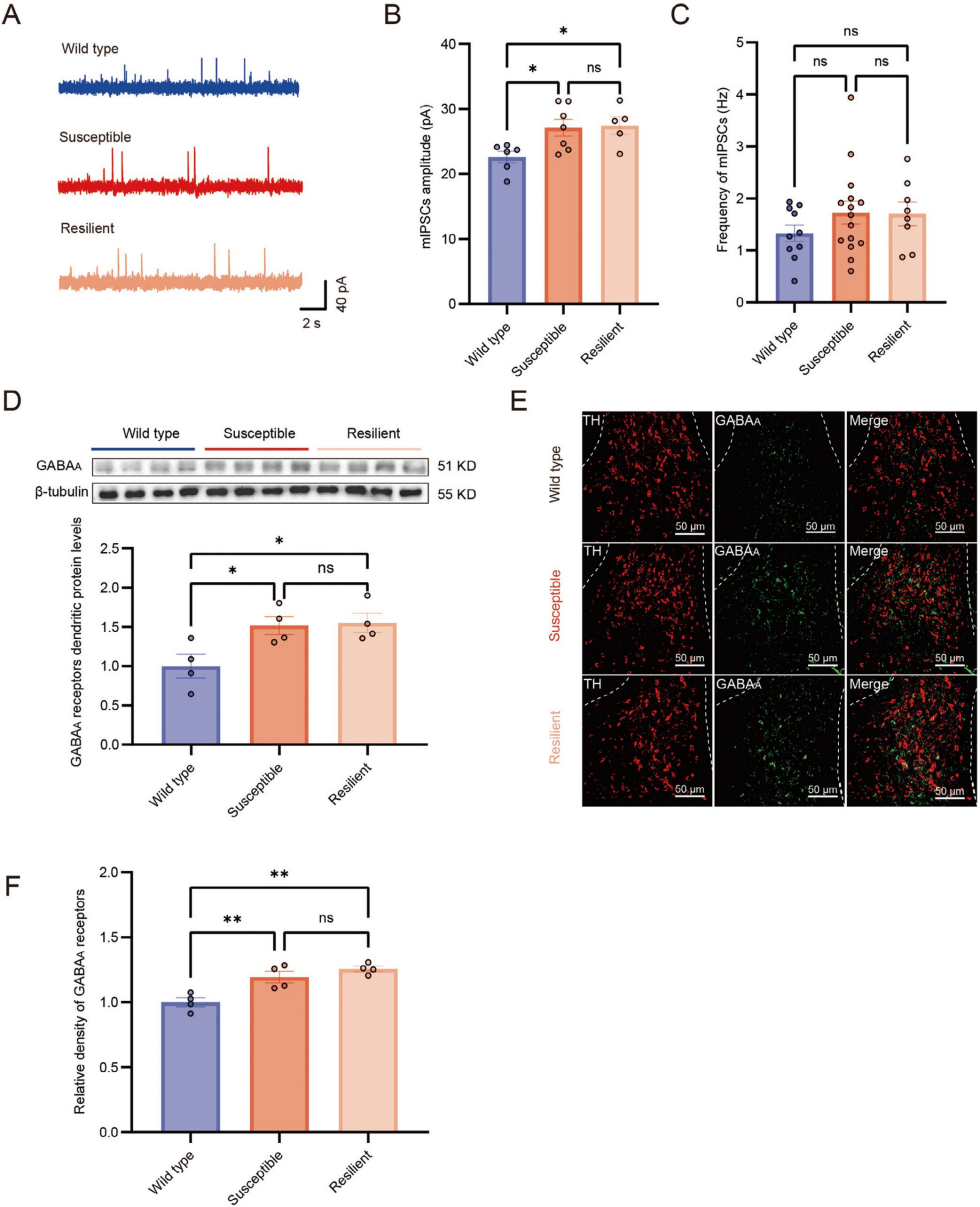
GABA_A_ receptors increased in VTA DA neurons of susceptible mice after CSDS. (A) Representative recording traces from VTA DA neurons showing mIPSCs from wild type, susceptible and resilient mice. (B) The mIPSCs amplitude of wild type (blue), susceptible (red) and resilient (light red) mice (One‐way ANOVA; wild type vs. susceptible: *P* = 0.02; susceptible vs. resilient: *P* = 0.86; wild type vs. resilient: *P* = 0.02; wild type: *N* = 6; susceptible: *N* = 7; resilient: *N* = 5). (C) The mIPSCs frequency of wild type (blue), susceptible (red) and resilient (light red) mice (One‐way ANOVA; wild type vs. susceptible: *P* = 0.18; susceptible vs. resilient: *P* = 0.94; wild type vs. resilient: *P* = 0.28; wild type: *N* = 10; susceptible: *N* = 15; resilient: *N* = 8). (D) GABA_A_ receptors dendritic protein level of VTA was measured using Western blot (One‐way ANOVA; wild type vs. susceptible: *P* = 0.02; susceptible vs. resilient: *P* = 0.86; wild type vs. resilient: *P* = 0.02; wild type: *N* = 4; susceptible: *N* = 4; resilient: *N* = 4). (E) Representative immunofluorescence images of the colocalization of TH (red) and GABA_A_ receptors (green) in the VTA of the wild type, susceptible and resilient mice. Use 20× objectives. TH, tyrosine hydroxylase. Scale bar, 50 μm. (F) Relative density analysis of GABA_A_ receptors in wild type, susceptible and resilient mice. (One‐way ANOVA; wild type vs. susceptible: *P* = 0.003; susceptible vs. resilient: *P* = 0.235; wild type vs. resilient: *P* = 0.0005; wild type: *N* = 4; susceptible: *N* = 4; resilient: *N* = 4). mIPSCs were measured at +30 mV. Data are expressed as mean ± SEM; *p < 0.05, **p < 0.01, ***p < 0.001, ****p < 0.0001, ns means no significant.

To investigate the effect of GABA_A_ receptor modulation on the excitability of VTA DA neurons in socially stressed mice, we conducted firing recordings of DA neurons both in vitro and in vivo. First, the inhibitory effect of muscimol was evaluated, and the Hill equation was used to fit the dose–response relationship of the firing frequency of VTA DA neurons projecting to the NAc, yielding an IC50 value of 1.379 μM (Figure 2A), which was used in subsequent experiments. Representative electrophysiological traces recorded in vitro and in vivo are presented (Figure S3A,B). Next, muscimol was applied to VTA DA neurons in vitro in wild type mice, resulting in decreased spontaneous firing frequency, which was reversed by treatment both with muscimol and bicuculline (Figure 3C). The spontaneous firing frequency of VTA DA neurons increased after treatment with bicuculline but remained unchanged when both bicuculline and muscimol were applied together (Figure 3D) in vitro in wild type mice. We also analyzed both the firing frequency and burst firing properties in vivo using single‐unit recording in wild type mice. Consistent with the in vitro recordings, muscimol decreased the firing frequency of VTA DA neurons, and this effect was reversed by bicuculline (Figure 3E). By contrast, bicuculline increased the firing frequency, with no further change after co‐treatment with muscimol and bicuculline (Figure 3F). Similarly, muscimol reduced the percentage of spikes in bursts, and this reduction was reversed by bicuculline (Figure 3G). In contrast, bicuculline increased the percentage of spikes in bursts, with no additional change following co‐treatment with muscimol and bicuculline (Figure 3H).

Subsequently, the effect of muscimol on the firing of VTA DA neurons was further assessed using cell‐attached recordings in vitro in both wild type and susceptible mice (Figure 2B). The spontaneous firing frequency of VTA DA neurons in vitro decreased after muscimol treatment, and this effect was reversed by treatment both with muscimol and bicuculline in wild type and susceptible mice (Figure 2C,D). However, comparison of brain slices from wild type and susceptible mice revealed that the decrease in spontaneous firing frequency of VTA DA neurons after muscimol treatment was greater in susceptible mice than in wild type mice (Figure 2E). Next, we examined the firing frequency and the percentage of spikes in bursts of VTA DA neurons using single‐unit recording in vivo in wild type and susceptible mice (Figure 2F). The firing frequency of VTA DA neurons was decreased after muscimol treatment and reversed by co‐treatment with muscimol and bicuculline in both wild type (Figure 2G) and susceptible mice (Figure 2H). Moreover, the muscimol‐induced reduction in firing frequency was greater in susceptible mice than in wild type mice (Figure 2I). Similarly, the percentage of spikes in bursts was reduced by muscimol and restored upon co‐treatment with muscimol and bicuculline in wild type (Figure 2J) and susceptible mice (Figure 2K). Notably, the muscimol‐induced decrease in the percentage of spikes in bursts was also greater in susceptible mice than in wild type mice (Figure 2L). These results suggest that postsynaptic GABA_A_ receptors in the VTA modulate the excitability of VTA DA neurons projecting to the NAc, and that this modulation is significantly affected by CSDS in susceptible mice. Furthermore, the burst firing of VTA DA neurons in vivo is also significantly influenced by modulation of these GABA_A_ receptors.

### 3.3 GABA_A_ Receptors Increased in VTA DA Neurons of Susceptible Mice After CSDS

To explore the mechanism underlying the altered firing patterns of DA neurons in the VTA following CSDS, we recorded mIPSCs from VTA DA neurons using whole‐cell voltage‐clamp in wild type and susceptible mice (Figure 3A). We found that the amplitude of mIPSCs in VTA DA neurons of susceptible mice was higher than that in wild type mice (Figure 3B). However, the frequency of mIPSCs did not differ significantly between susceptible and wild type mice (Figure 3C). These findings suggest that postsynaptic GABA_A_ receptors on VTA DA neurons are upregulated following CSDS in susceptible mice, while presynaptic GABA release remains unchanged. To further verify changes in the GABA system in the VTA after CSDS, we performed western blot and immunofluorescence analyses to examine GABA_A_ receptor expression on dendrites. Quantitative analysis of dendritic proteins from the VTA revealed that the GABA_A_ receptor content in susceptible mice was significantly higher than in wild type mice (Figure 3D). Additionally, the fluorescence density of GABA_A_ receptors on dendrites around the cell bodies of DA neurons in the VTA was higher in susceptible mice than in wild type mice (Figure 3E,F). To extend these findings, we also examined resilient mice. Resilient mice exhibited GABAergic adaptations—including increased mIPSCs amplitude (Figure 3B,C), elevated GABA_A_ receptor protein levels (Figure 3D), and enhanced dendritic receptor density in the VTA (Figure 3E,F)—that were similar to those observed in susceptible mice.

Although both susceptible and resilient mice showed elevated GABA_A_ receptor levels in the VTA after CSDS, the observed divergent outcomes—specifically, the markedly heightened excitability of VTA DA neurons and the distinct social avoidance behavior unique to susceptible mice—lead us to subsequently focus on elucidating the mechanism by which DA neuron hyperexcitability drives stress‐related social behaviors in this specific phenotype.

### 3.4 Optogenetic Activation of GABA Neuron Terminals From RMTg Reduced the Excitability of VTA DA Neurons in Susceptible Mice

**FIGURE 4:**
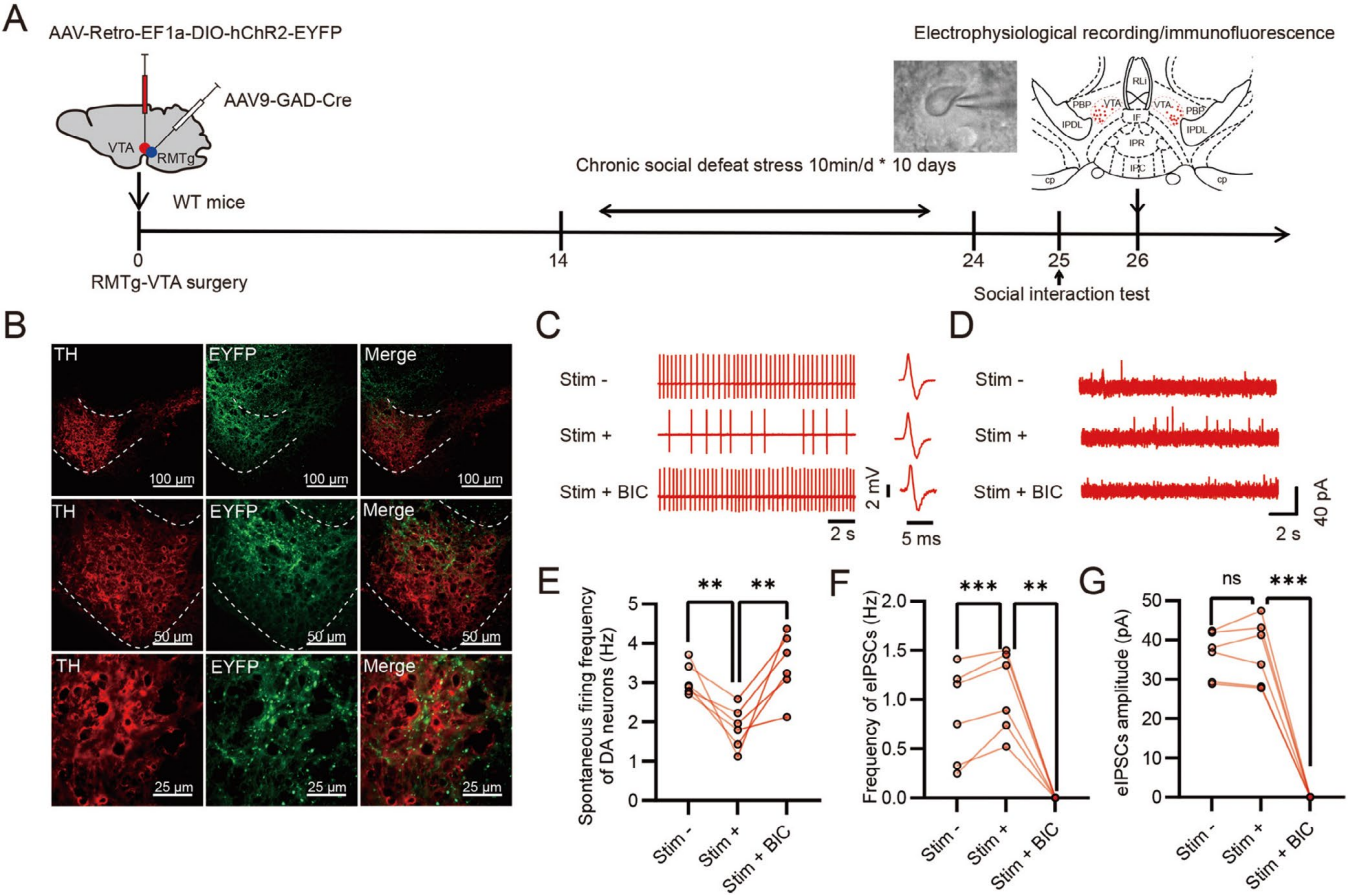
Optogenetic activation of GABA neurons in RMTg reduced the excitability of VTA DA neurons in susceptible mice. (A) Timeline for the establishment of RMTg‐VTA surgery, the CSDS model, the social interaction (SI) test, immunofluorescence, and electrophysiological recordings. The left panel on the upper right shows the placement of the glass electrode and the right panel illustrates a coronal midbrain slice indicating the locations of the recorded DA neurons (red dots). (B) Representative immunofluorescence images of the colocalization of ChR2‐EYFP‐expressing DA neurons (green) and TH (red) in the VTA, indicating that DA neurons in the VTA receive projections from GABA neurons in the RMTg. Use 10× objective lenses (top), 20× objective lenses (middle) and 40× objective lenses (bottom). TH, tyrosine hydroxylase. Scale bars, 100 μm, 50 μm, 25 μm. (C) Representative firing of DA neurons from susceptible mice before and after optogenetic activation and without or with BIC treatment in the VTA. (D) Representative recording traces from DA neurons showing mIPSCs before and after optogenetic activation in the VTA from susceptible mice. (E) Spontaneous firing of DA neurons in susceptible mice before and after optogenetic activation and without or with BIC treatment (Paired sample *t* test, respectively, no stimulation vs. stimulation: *T* = 4.056, *p* = 0.01, stimulation vs. stimulation + BIC: *T* = −4.054, *p* = 0.01, *n* = 6 cells from 5 susceptible mice) in the VTA. (F) The frequency (Paired sample *t* test, respectively, no stimulation vs. stimulation: *T* = −7.80, *p* = 0.0006, stimulation vs. stimulation + BIC: *T* = 6.363, *p* = 0.0014, *n* = 6 cells from 5 susceptible mice) of eIPSCs recorded from DA neurons before and after optogenetic activation and without or with BIC treatment in the VTA. (G) The amplitude (Paired sample *t* test, respectively, no stimulation vs. stimulation: *T* = −0.568, *p* = 0.595, stimulation vs. stimulation + BIC: *T* = 11.01, *p* = 0.00012, *n* = 6 cells from 5 susceptible mice) of eIPSCs recorded from DA neurons before and after optogenetic activation and without or with BIC treatment in the VTA. eIPSCs were measured at +30 mV. Data are expressed as mean ± SEM; *p < 0.05, **p < 0.01, ***p < 0.001, ****p < 0.0001, ns means no significant.

Previous research has shown that VTA DA neurons receive direct monosynaptic GABA inputs from the RMTg. To selectively activate GABA neurons in the RMTg region, AAV9‐GAD‐Cre was injected into the RMTg region, and AAV‐Retro‐EF1a‐DIO‐hChR2‐EYFP was injected into the VTA region. After waiting for 3 weeks, the virus was fully expressed. Brain slices were subjected to TH immunofluorescence staining or in vitro electrophysiological experiments (Figure 4A). The results indicated that the synaptic terminals of GABA neurons in the RMTg were localized around DA neurons in the VTA (Figure 4B). All DA neurons in the VTA in vitro were identified during recordings based on their electrophysiological properties (Figure 4C). After chronic social defeat stress, the spontaneous firing and eIPSCs of VTA DA neurons in susceptible mice were recorded using cell‐attached patch‐clamp and whole‐cell voltage‐clamp techniques (Figure 4C,D). Following optogenetic (473 nm) activation of GABA neuron synaptic terminals in the VTA, the spontaneous firing frequency of DA neurons in susceptible mice was significantly reduced, but this effect was reversed by the addition of bicuculline (Figure 4E). The eIPSCs in VTA DA neurons of susceptible mice were also recorded following optogenetic (473 nm) activation of GABA neuron terminals in the VTA. The frequency of eIPSCs in DA neurons of the VTA in susceptible mice significantly increased (Figure 4F), while the amplitude of eIPSCs remained unchanged (Figure 4G). The eIPSCs in susceptible mice were abolished following the addition of bicuculline (Figure 4F,G). These results indicate that specific activation of GABA neurons in the RMTg region enhances GABA release in the VTA, subsequently inhibiting the excitability of DA neurons through GABA_A_ receptors.

### 3.5 Activation of GABA Neurons in the RMTg Region Reversed Social Stress Behavior in Susceptible Mice

**FIGURE 5:**
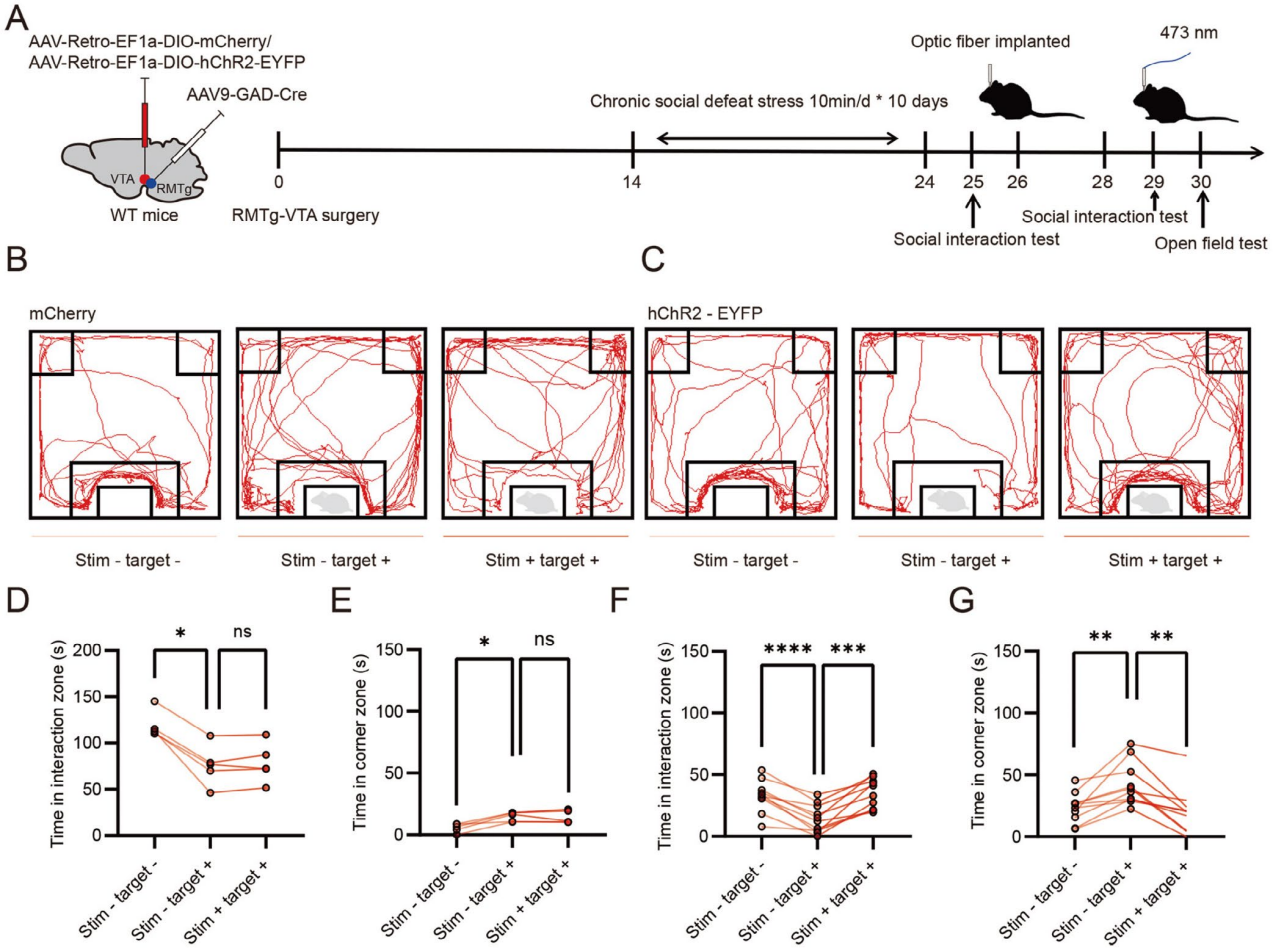
Activation of GABA neurons in the RMTg region reversed social stress behavior in susceptible mice. (A) Timeline for the establishment of RMTg‐VTA surgery, the CSDS model, social interaction (SI) test and optogenetics. (B) and (C) Representative traces of susceptible mice during the social interaction test which were injected with AAV9‐GAD‐Cre and AAV‐Retro‐EF1a‐DIO‐hChR2‐EYFP/AAV‐Retro‐EF1a‐DIO‐mCherry, before and after optogenetic activation in the VTA. (D) Time spent in the social interaction zone of susceptible mice injected with AAV‐Retro‐EF1a‐DIO‐mCherry and AAV9‐GAD‐Cre (Wilcoxon's symbolic rank test, no stimulation + no target vs. no stimulation + target: *P* = 0.043, Paired sample *t* test, no stimulation + target vs. stimulation + target: *T* = −1.181, *p* = 0.303, *n* = 5 susceptible mice). (E) Time spent in the corner zone of susceptible mice injected with AAV‐Retro‐EF1a‐DIO‐mCherry and AAV9‐GAD‐Cre (Wilcoxon's symbolic rank test, respectively, no stimulation + no target vs. no stimulation + target: *P* = 0.043, no stimulation + target vs. stimulation + target: *P* = 0.893, *n* = 5 susceptible mice). (F) Time spent in the social interaction zone of susceptible mice injected with AAV‐Retro‐EF1a‐DIO‐hChR2‐EYFP and AAV9‐GAD‐Cre (Paired sample *t* test, respectively, no stimulation + no target vs. no stimulation + target: *T* = 6.87, *p* < 0.0001, no stimulation + target vs. stimulation + target: *T* = −6.054, *p* = 0.0002, *n* = 10 susceptible mice). (G) Time spent in the corner zone of susceptible mice injected with AAV‐Retro‐EF1a‐DIO‐hChR2‐EYFP and AAV9‐GAD‐Cre (Paired sample *t* test, respectively, no stimulation + no target vs. no stimulation + target: *T* = −4.099, *p* = 0.003, Wilcoxon's symbolic rank test, no stimulation + target vs. stimulation + target: *P* = 0.005, *n* = 10 susceptible mice). *p < 0.05, **p < 0.01, ***p < 0.001, ****p < 0.0001, ns means no significant.

To investigate the effects of optogenetic activation of RMTg GABAergic neurons projecting to VTA dopaminergic neurons on social behavior in mice subjected to CSDS, we conducted behavioral assays in susceptible mice. AAV constructs (AAV9‐GAD‐Cre and AAV‐Retro‐EF1a‐DIO‐hChR2‐EYFP/AAV‐Retro‐EF1a‐DIO‐mCherry) were injected into the RMTg and VTA brain regions of mice, respectively. Two weeks later, mice suffering from a 10‐day CSDS paradigm were identified as susceptible based on their social interaction ratios (Figure 5A). Optical fibers were implanted in the VTA of susceptible mice for targeted stimulation to selectively activate the endings of GABA neurons in the RMTg region. Representative tracking plots of control virus‐injected and hChR2‐injected mice during the social interaction test are shown (Figure 5B,C). In susceptible mice injected with the control virus, light stimulation (473 nm) produced no significant changes in behavioral responses, as reflected by the time spent in the social interaction zone and the corner zone (Figure 5D,E). In contrast, optogenetic stimulation of RMTg GABAergic neurons in susceptible mice injected with hChR2 virus significantly increased the time spent in the social interaction zone and decreased the time spent in the corner zone (Figure 5F,G), indicating a reversal of stress‐induced social avoidance behavior.

Furthermore, to rule out potential confounding effects of optogenetic activation on locomotor activity in social behavior tests, we performed the open field test (Figure 4A). The results showed no significant differences in either the time spent in the center zone or the total distance traveled before versus after optogenetic activation of GABAergic neurons in the RMTg region (Figure S4B,C). These results suggest that selective activation of RMTg GABAergic neurons effectively modulates VTA DA neuron excitability and reverses stress‐induced social withdrawal in CSDS‐susceptible mice.

## 4 Discussion

Our study demonstrated a significant increase in GABA_A_ receptor density in the VTA of susceptible mice following CSDS. Pharmacological activation of GABA_A_ receptors reduced the burst excitability of VTA DA neurons after social stress and increased social interaction time in susceptible mice.

The VTA DA system plays a central and complex role in stress responses, with its functional changes exhibiting significant variations depending on the nature of stressors [29]. Specifically, in the chronic unpredictable mild stress (CUMS) model, susceptible mice demonstrate decreased activity of VTA DA neurons [30, 31], and correspondingly, activating these neurons can effectively ameliorate their depression‐like behaviors [30]. In contrast, in the CSDS model, susceptible mice show markedly increased activity of VTA DA neurons, which is highly correlated with reduced social interaction time. Suppressing this hyperactivity can significantly alleviate depression‐like behaviors [18, 32]. Previous research has identified the VTA–NAc pathway as a critical determinant of susceptibility versus resilience to repeated social stress [33], with increased burst firing of DA neurons specifically observed in susceptible mice. In our study, VTA DA neurons were identified using retrobeads injected into the NAc in combination with electrophysiological characterization. The labeled DA neurons projecting to the NAc exhibited similar electrophysiological properties in susceptible mice. Notably, direct modulation of DA neuron excitability led to changes in burst firing patterns. Burst firing of VTA DA neurons has been shown to play a pivotal role in the development of social stress‐induced behavioral alterations [21]. Consistent with this, our findings revealed that pharmacological inhibition of DA neuron burst firing via muscimol treatment in the VTA significantly increased social interaction time in susceptible mice. Collectively, these findings suggest that modulation of GABA_A_ receptors on VTA DA neurons represents a potential therapeutic target for reversing social stress‐induced behavioral deficits.

GABA_A_ receptors are ligand‐gated chloride channels that mediate fast synaptic inhibition by reducing neuronal excitability [34]. Previous studies have implicated GABA_A_ receptor‐mediated inhibition of VTA DA neurons in behaviors related to alcohol‐seeking [35, 36], which aligns with our findings. Our results indicated that the expression of GABA_A_ receptors on dendritic spines in the VTA is increased in stress‐susceptible mice. DA neurons account for approximately 70% of neurons in this region and play a critical role in the development of depression‐like behaviors.

However, an intriguing finding was that although GABA_A_ receptor expression was increased on VTA DA neurons of resilient mice, the spontaneous firing frequency of these neurons was significantly lower than in susceptible mice. This seemingly paradoxical result suggested that the regulation of neuronal excitability in resilient mice likely involves a more complex, integrated mechanism beyond simple GABAergic inhibition. This aligns with previous studies indicating that resilience does not arise from passive resistance of a single pathway but rather from active, multi‐layered homeostatic remodeling of ion channels [28, 33]. Notably, while excitatory inputs (such as the Ih current) were enhanced in VTA DA neurons of resilient mice, there is a specific upregulation of potassium channel function, including KCNQ channels, which generates strong compensatory inhibitory currents [28, 37]. Therefore, the observed upregulation of GABA_A_ receptors in this study may synergize with this intrinsic enhancement of potassium channels, together forming a more stable excitation‐inhibition balance network. This ultimately fine‐tunes neuronal activity to a normal physiological range, which may be key to the sustained maintenance of the resilient phenotype.

In addition, both in vitro and in vivo electrophysiological recordings showed that DA neurons in the VTA of susceptible mice exhibited larger mIPSCs amplitude and heightened sensitivity to GABA_A_ receptor agonists. These findings collectively indicate an upregulation of GABA_A_ receptor expression on the dendritic spines of DA neurons in susceptible mice. One possible explanation is that social stress increases the excitability of VTA DA neurons, which in turn triggers a compensatory negative feedback mechanism. Consistent with our previous findings, ion channels that regulate DA neuron excitability are also altered under stress conditions. However, the underlying mechanisms governing these receptor and ion channel changes require further investigation. In addition to GABA_A_ receptors, the excitability of VTA DA neurons is regulated by multiple receptor types, including GABA_B_ and glutamate receptors. GABA_B_ receptors are G‐protein‐coupled receptors that activate G protein‐coupled inwardly‐rectifying potassium (Kir) channels, leading to slow IPSCs [34, 38]. Recent studies have shown that GABA_B_ receptors on VTA DA neurons indirectly regulate Kir3.2 channel activity via G‐protein signaling, thereby modulating DA neuron excitability and influencing social stress‐related behaviors [39], and influence animal social stress behaviors [40]. These findings suggest that VTA DA neuron excitability may be differentially regulated by GABAergic inputs through distinct receptor subtypes, a hypothesis that warrants further investigation in future studies.

Beyond intrinsic receptor regulation, VTA DA neuron activity is also modulated by inhibitory GABAergic inputs from the NAc [24] and RMTg [41]. GABA neurons in the medial or lateral shell of the NAc exert direct inhibition on VTA DA neurons or disinhibit the DA neurons by synapsing onto VTA GABA neurons. On the other hand, the DA neurons in the VTA were also strongly innervated by GABAergic neurons in the RMTg, which play a central role in balancing reward and aversion [42]. For example, GABAergic neurons in the RMTg inhibited VTA dopaminergic neurons through receiving glutamatergic projections from the lateral habenula (LHb) [43]. Recent research showed an imbalance in the excitatory–inhibitory inputs to the RMTg‐projecting LHb neurons in susceptible mice, suggesting that CSDS may also alter presynaptic transmission at LHb neurons projecting to RMTg [41]. In our study, optogenetic activation of GABAergic projections from the RMTg to the VTA reduced the excitability of VTA DA neurons and alleviated social defeat‐induced behavioral deficits. Moving forward, we aim to explore projections from additional brain regions to VTA DA neurons and their roles in modulating social stress‐related behaviors.

## 5 Conclusion

In summary, our study identified an increase in GABA_A_ receptor density and function in the VTA of CSDS‐susceptible mice, which was associated with enhanced mIPSCs and increased burst firing of DA neurons. Furthermore, pharmacological modulation of GABA_A_ receptors using muscimol, as well as optogenetic activation of RMTg GABAergic neurons, effectively reduced VTA DA neuron excitability and reversed social stress‐induced behavioral deficits. These findings highlight the crucial role of GABAergic modulation within the VTA in stress‐related behavioral abnormalities. Importantly, our results suggest that targeting the GABA_A_ receptor pathway and GABAergic signaling in the VTA may represent a promising therapeutic strategy for neuropsychiatric disorders such as anxiety and depression.

## Author Contributions

**Guang‐Yue Ma:** designed the research, performed electrophysiological experiments in vivo and in vitro. **Jin‐Zhu Zhuang:** designed the research, performed electrophysiological experiments in vivo and in vitro. **Shu‐Feng Li:** performed behavioral and morphological experiments. **Yu‐E Zhang:** performed behavioral experiments. **Di Zhang:** identified behavioral model characteristics. **Zhen Peng:** performed behavioral experiments. **Liu Yang:** performed animal feeding and surgery. **Zhang Cao:** provided experimental animal model. **Xin Xie:** designed and supervised the study and analyzed the data. **Rong Jiang:** consulted on the project and edited the manuscript. **Hui Sun:** designed and supervised the study, analyzed the data, and wrote the manuscript.

## Funding

The work is funded by the National Natural Science Foundation of China (81971281), the National Natural Science Foundation of China (82301726), the Natural Science Foundation of Shandong Province (ZR2022QH087), and the Project of Shandong Province Higher Educational Science and Technology Program (J18KA165).

## Ethics Statement

The animal protocols were approved by the Animal Use and Care Committee of Binzhou Medical University (Protocol # SYXK‐220130020‐03). The experiments involving mice were in accordance with the National Institute of Health Guide for the Care and Use of Laboratory Animals (NIH Publications No. 80–23).

## Consent

The authors have nothing to report.

## Conflicts of Interest

The authors declare no conflicts of interest.

## Supporting information


**Figure S1:** The frequency of VTA DA neurons firing in the VTA was increased in susceptible mice. (A) Timeline for the establishment of the VTA‐NAc surgery, CSDS model, social interaction (SI) test, immunofluorescence and electrophysiological recordings. The left panel on the upper right illustrates a coronal midbrain slice indicating the locations of the recorded DA neurons (red dots) and the right panel shows the placement of the glass electrode. (B) Representative immunofluorescence images of the colocalization of retrobeads (red) and DA neurons (green) in CAG‐GFP × DAT‐Ires‐Cre mice. Use 20× objectives. Red retrobeads, red fluorescent retrograde marking bead. Scale bar, 50 μm. (C) and (D) Representative VTA DA neurons spontaneous firing in vitro and vivo. (E) Spontaneous firing frequency of VTA DA neurons in vitro from wild type (blue), susceptible (red) and resilient (light red) mice (One‐way ANOVA; wild type versus susceptible: *p* = 0.001; susceptible versus resilient: *p* = 0.047; wild type vs. resilient: *p* = 0.072; wild type: *n* = 7); susceptible: *n* = 6; resilient: *n* = 5. (F) The firing frequency of VTA DA neurons recorded in vivo from wild type (blue), susceptible (red) and resilient (light red) mice (One‐way ANOVA; wild type vs. susceptible: *p* = 0.008; susceptible vs. resilient: *p* = 0.015; wild type vs. resilient: *p* = 0.763; wild type: *n* = 7; susceptible: *n* = 7; resilient: *n* = 7). (G) The burst firing (percentage of spikes in bursts) of VTA DA neurons in vivo from wild type (blue), susceptible (red) and resilient (light red) mice (One‐way ANOVA; wild type vs. susceptible: *p* = 0.001; susceptible vs. resilient: *p* = 0.003; wild type versus resilient: *p* = 0.407; wild type: *n* = 7); susceptible: *n* = 7; resilient: *n* = 7. Data are expressed as mean ± SEM; *p < 0.05, **p < 0.01, ***p < 0.001, ****p < 0.0001, ns means no significant.
**Figure S2:** Inhibition of GABAA receptors failed to influence social stress behaviors in susceptible mice. (A) Representative traces of susceptible mice during the social interaction test treated with vehicle, BIC or/and MUS. (B) Time spent in the social interaction zone of susceptible mice treated with vehicle, BIC or/and MUS (One‐way ANOVA; Vehicle vs. BIC: *p* = 0.703; BIC vs. BIC + MUS: *p* = 0.418; Vehicle vs. BIC + MUS: *p* = 0.664; Vehicle: *n* = 8; BIC: *n* = 8; BIC + MUS: *n* = 8). (C) Time spent in the corner zone by susceptible mice treated with vehicle, MUS or/and BIC (One‐way ANOVA; Vehicle vs. BIC: *p* = 0.374; BIC vs. BIC + MUS: *p* = 0.394; Vehicle vs. BIC + MUS: *p* = 0.969; Vehicle: *n* = 8; BIC: *n* = 8; BIC + MUS: *n* = 8). Data are expressed as mean ± SEM; *p < 0.05, **p < 0.01, ***p < 0.001, ****p < 0.0001, ns means no significant.
**Figure S3:** Muscimol decreased the excitability of VTA DA neurons. (A) Typical spontaneous firing of VTA DA neurons from brain slices. (B) The time course of the firing frequency of VTA DA neurons with MUS or/and BIC from wild type mice in vivo. Typical recordings and action potential waveforms are shown. (C) Spontaneous firing frequency of VTA DA neurons decreased in wild type mice with muscimol (1.379 μM) (MUS) or/and bicuculline (1 μM) (BIC) in vitro (*n* = 5 cells from 5 wild type mice, Paired sample *t* test: Vehicle vs. MUS: *t* = 5.973, *p* = 0.004, Wilcoxon's symbolic rank test, MUS vs. MUS + BIC: *p* = 0.043). (D) Spontaneous firing frequency of VTA DA neurons increased in wild type mice with BIC or/and MUS in vitro (*n* = 11 cells from 5 wild type mice, Wilcoxon's symbolic rank test: Vehicle vs. MUS: *p* = 0.003, MUS vs. MUS + BIC: *p* = 0.266). (E) and (F) Effect of MUS (*n* = 9 cells from 5 wild type mice, Paired sample *t* test: Vehicle vs. MUS: *t* = 10.033, *p* < 0.0001, MUS vs. MUS + BIC: *t* = −16.764, *p* < 0.0001) or/and BIC (*n* = 8 cells from 5 wild type mice, Paired sample *t* test: Vehicle vs. MUS: *t* = −3.768, *p* = 0.007, MUS vs. MUS + BIC: *t* = 0.326, *p* = 0.754) on the firing frequency of VTA DA neurons from wild type mice in vivo. (G) and (H) Effects of MUS (*n* = 9 cells from 5 wild type mice, Wilcoxon's symbolic rank test: Vehicle vs. MUS: *p* = 0.008, MUS vs. MUS + BIC: *p* = 0.008) or/and BIC (*n* = 8 cells from 5 wild type mice, Paired sample *t* test: Vehicle vs. MUS: *t* = −10.466, *p* < 0.0001, MUS vs. MUS + BIC: *t* = −1.971, *p* = 0.089) on the percentage of spikes in bursts in vivo. Data are expressed as mean ± SEM; *p < 0.05, **p < 0.01, ***p < 0.001, ****p < 0.0001, ns means no significant.
**Figure S4:** Activation of GABA neurons in the RMTg region did not alter motor function in susceptible mice. (A) Representative traces of susceptible mice during the open field test which were injected with AAV9‐GAD‐Cre and AAV‐Retro‐EF1a‐DIO‐hChR2‐EYFP. (B) Time spent in the centre area of susceptible mice in open field (Paired sample *t* test, no stimulation vs. stimulation: *t* = 0.984, *p* = 0.351, *n* = 10 susceptible mice). (C) Total distance in open field of susceptible mice (Paired sample *t* test, no stimulation vs. stimulation: *t* = 1.469, *p* = 0.176, *n* = 10 susceptible mice). Data are expressed as mean ± SEM; *p < 0.05, **p < 0.01, ***p < 0.001, ****p < 0.0001, ns means no significant.

## Data Availability

The data that support the findings of this study are available on request from the corresponding author. The data are not publicly available due to privacy or ethical restrictions.
