# Supplementary material for: GABAergic Neuron Activation in the RMTg‐VTA Pathway Modulates Dopaminergic Neuron Excitability and Social Stress Susceptibility in Male Mice
Source: CNS Neurosci Ther. 2026 Apr 1;32(4):e70855. doi: 10.1002/cns.70855 (PMC13045366; doi:10.1002/cns.70855)
Supplement: Supplementary file 1 — Figure S1: The frequency of VTA DA neurons firing in the VTA was increased in susceptible mice. (A) Timeline for the establishment of the VTA‐NAc surgery, CSDS model, social interaction (SI) test, immunofluorescence and electrophysiological recordings. The left panel on the upper right illustrates a coronal midbrain slice indicating the locations of the recorded DA neurons (red dots) and the right panel shows the placement of the glass electrode. (B) Representative immunofluorescence images of the colocalization of retrobeads (red) and DA neurons (green) in CAG‐GFP × DAT‐Ires‐Cre mice. Use 20× objectives. Red retrobeads, red fluorescent retrograde marking bead. Scale bar, 50 μm. (C) and (D) Representative VTA DA neurons spontaneous firing in vitro and vivo. (E) Spontaneous firing frequency of VTA DA neurons in vitro from wild type (blue), susceptible (red) and resilient (light red) mice (One‐way ANOVA; wild type versus susceptible: p = 0.001; susceptible versus resilient: p = 0.047; wild type vs. resilient: p = 0.072; wild type: n = 7); susceptible: n = 6; resilient: n = 5. (F) The firing frequency of VTA DA neurons recorded in vivo from wild type (blue), susceptible (red) and resilient (light red) mice (One‐way ANOVA; wild type vs. susceptible: p = 0.008; susceptible vs. resilient: p = 0.015; wild type vs. resilient: p = 0.763; wild type: n = 7; susceptible: n = 7; resilient: n = 7). (G) The burst firing (percentage of spikes in bursts) of VTA DA neurons in vivo from wild type (blue), susceptible (red) and resilient (light red) mice (One‐way ANOVA; wild type vs. susceptible: p = 0.001; susceptible vs. resilient: p = 0.003; wild type versus resilient: p = 0.407; wild type: n = 7); susceptible: n = 7; resilient: n = 7. Data are expressed as mean ± SEM; *p < 0.05, **p < 0.01, ***p < 0.001, ****p < 0.0001, ns means no significant. Figure S2: Inhibition of GABAA receptors failed to influence social stress behaviors in susceptible mice. (A) Representative traces of susce [file CNS-32-e70855-s001.docx]

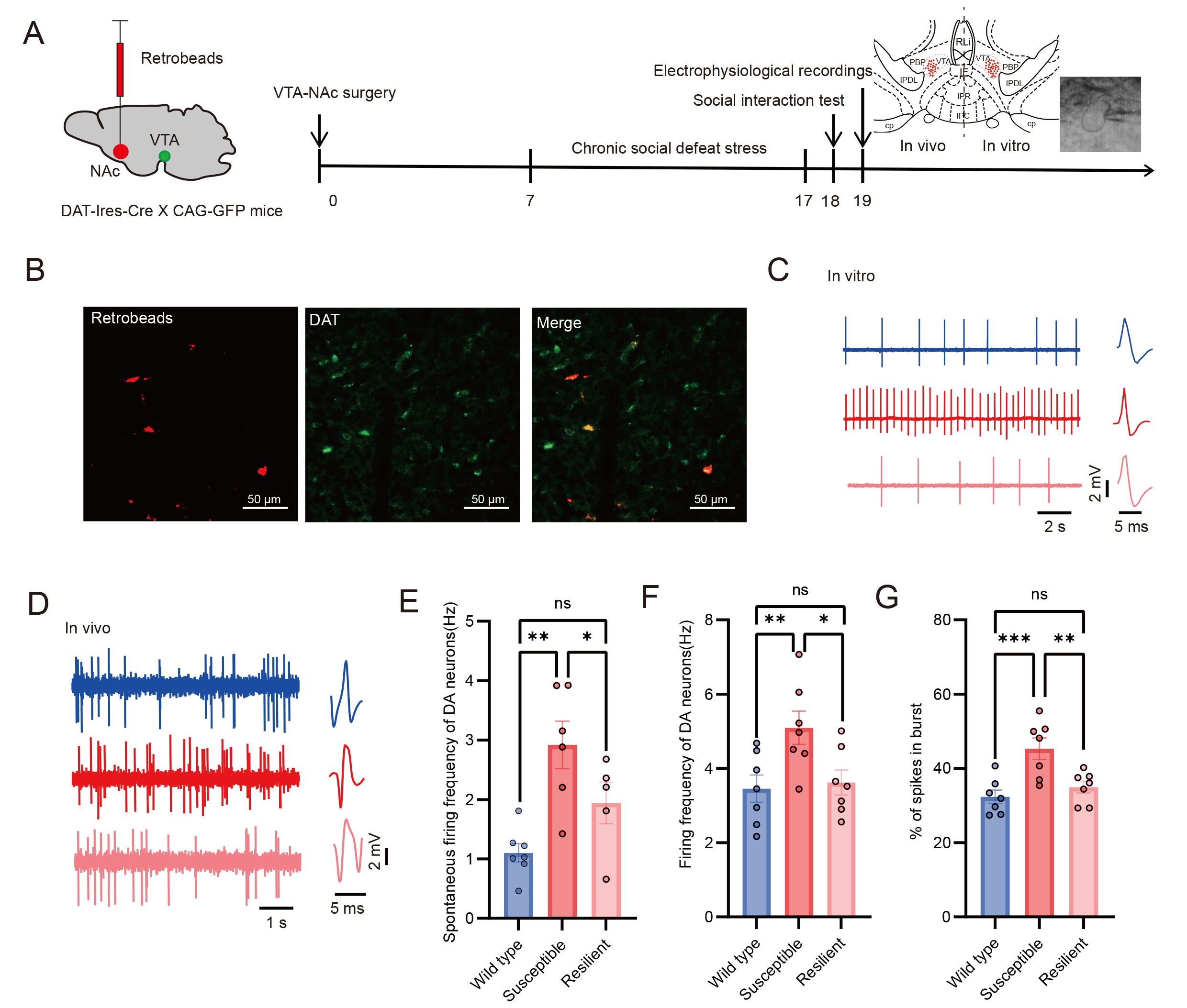


**Supplementary Fig. 1. The frequency of VTA DA neurons firing in the VTA was increased in susceptible mice.** (A) Timeline for the establishment of the VTA-NAc surgery, CSDS model, social interaction (SI) test, immunofluorescence and electrophysiological recordings. The left panel on the upper right illustrates a coronal midbrain slice indicating the locations of the recorded DA neurons (red dots) and the right panel shows the placement of the glass electrode. (B) Representative immunofluorescence images of the colocalization of retrobeads (red) and DA neurons (green) in CAG-GFP × DAT-Ires-Cre mice. Use 20x objectives. Red retrobeads, red fluorescent retrograde marking bead. Scale bar, 50 µm. (C) and (D) Representative VTA DA neurons spontaneous firing in vitro and vivo. (E) Spontaneous firing frequency of VTA DA neurons in vitro from wild type (blue), susceptible (red) and resilient (light red) mice One-way ANOVA; wild type vs. susceptible: *P* = 0.001; susceptible vs. resilient: *P* = 0.047; wild type vs. resilient: *P* = 0.072; wild type: n = 7; susceptible: n = 6; resilient: n = 5). (F) The firing frequency of VTA DA neurons recorded in vivo from wild type (blue), susceptible (red) and resilient (light red) mice One-way ANOVA; wild type vs. susceptible: *P* = 0.008; susceptible vs. resilient: *P* = 0.015; wild type vs. resilient: *P* = 0.763; wild type: n = 7; susceptible: n = 7; resilient: n = 7). (G) The burst firing (percentage of spikes in bursts) of VTA DA neurons in vivo from wild type (blue), susceptible (red) and resilient (light red) mice One-way ANOVA; wild type vs. susceptible: *P* = 0.001; susceptible vs. resilient: *P* = 0.003; wild type vs. resilient: *P* = 0.407; wild type: n = 7; susceptible: n = 7; resilient: n = 7). Data are expressed as mean ± SEM; **P* < 0.05, ***P* < 0.01, ****P* < 0.001, *****P* < 0.0001, ns means no significant.

**
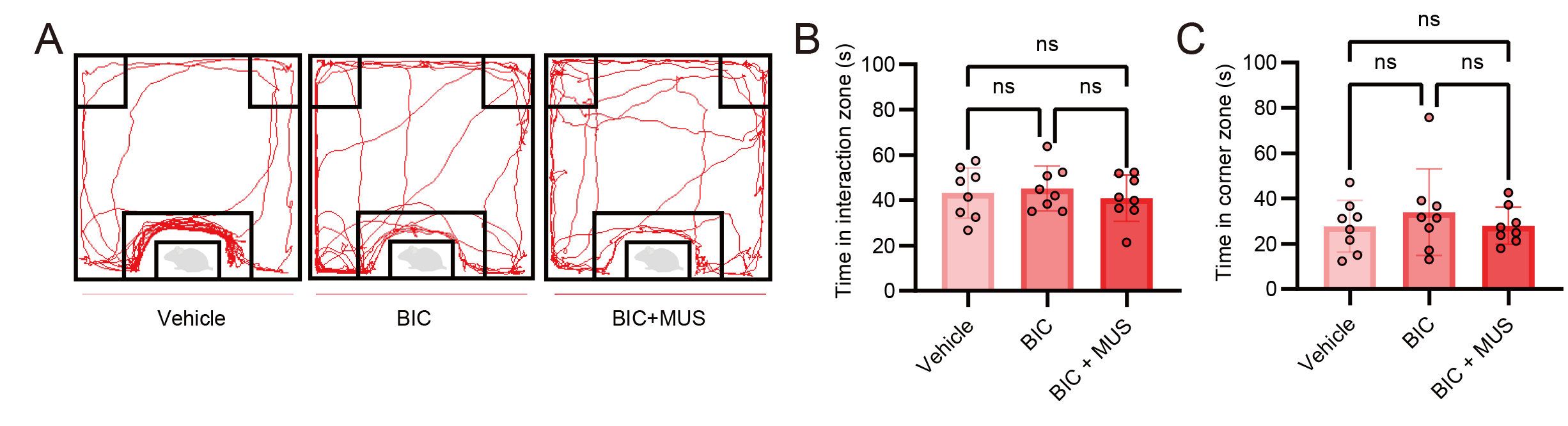
**

**Supplementary Fig. 2. Inhibition of GABA_A_ receptors failed to influence social stress behaviors in susceptible mice.** (A) Representative traces of susceptible mice during the social interaction test treated with vehicle, BIC or/and MUS. (B) Time spent in the social interaction zone of susceptible mice treated with vehicle, BIC or/and MUS (One-way ANOVA; Vehicle vs. BIC: *P* = 0.703; BIC vs. BIC + MUS: *P* = 0.418; Vehicle vs. BIC + MUS: *P* = 0.664; Vehicle: n = 8; BIC: n = 8; BIC + MUS: n = 8). (C) Time spent in the corner zone by susceptible mice treated with vehicle, MUS or/and BIC (One-way ANOVA; Vehicle vs. BIC: *P* = 0.374; BIC vs. BIC + MUS: *P* = 0.394; Vehicle vs. BIC + MUS: *P* = 0.969; Vehicle: n = 8; BIC: n = 8; BIC + MUS: n = 8). Data are expressed as mean ± SEM; **P* < 0.05, ***P* < 0.01, ****P* < 0.001, *****P* < 0.0001, ns means no significant.


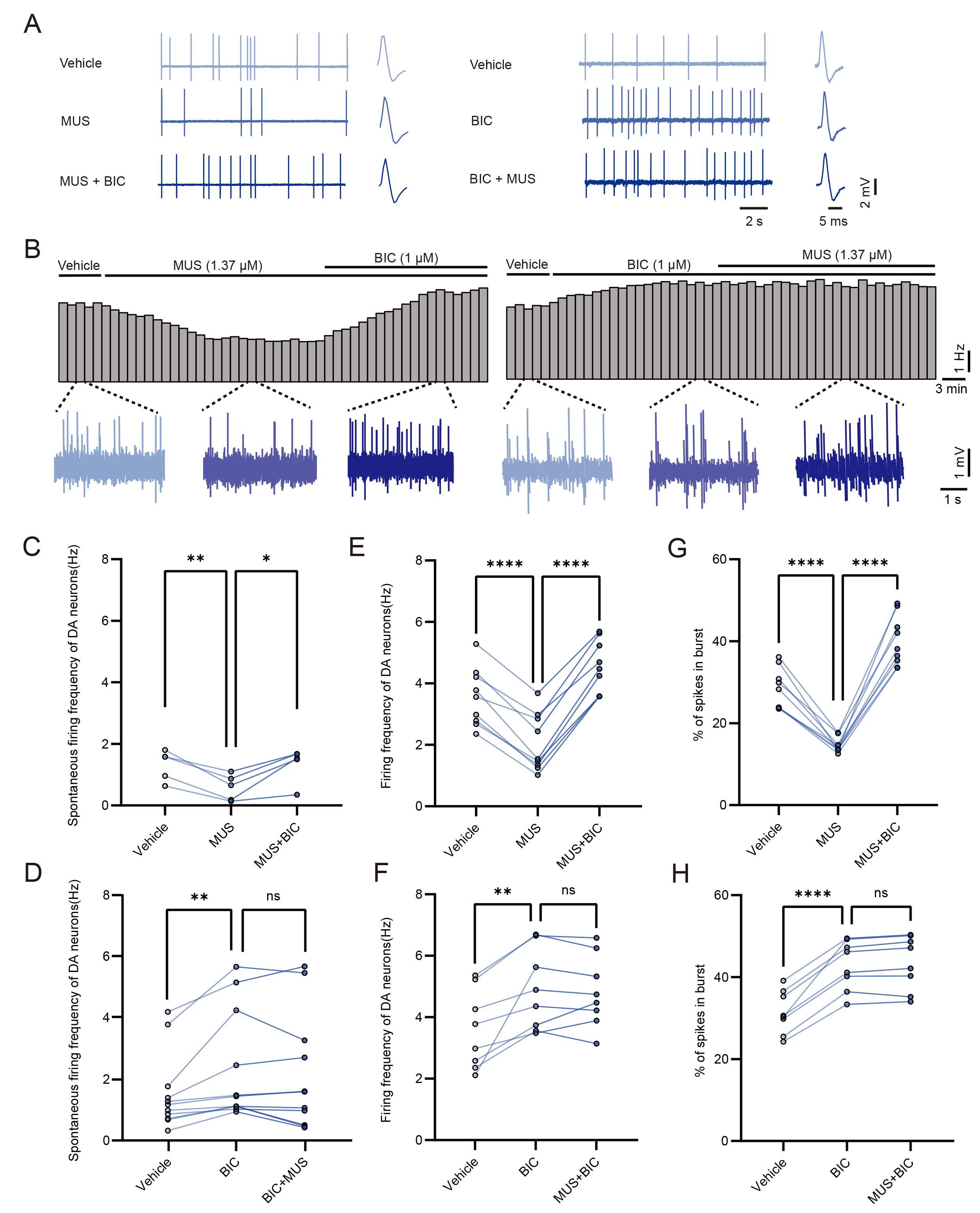


**Supplementary Fig. 3. Muscimol decreased the excitability of VTA DA neurons.** (A) Typical spontaneous firing of VTA DA neurons from brain slices. (B) The time course of the firing frequency of VTA DA neurons with MUS or/and BIC from wild type mice in vivo. Typical recordings and action potential waveforms are shown. (C) Spontaneous firing frequency of VTA DA neurons decreased in wild type mice with muscimol (1.379 μM) (MUS) or/and bicuculline (1 μM) (BIC) in vitro (n = 5 cells from 5 wild type mice, Paired sample t test: Vehicle vs. MUS: t = 5.973, *P* = 0.004, Wilcoxon's symbolic rank test, MUS vs. MUS + BIC: *P* = 0.043). (D) Spontaneous firing frequency of VTA DA neurons increased in wild type mice with BIC or/and MUS in vitro (n = 11 cells from 5 wild type mice, Wilcoxon's symbolic rank test: Vehicle vs. MUS: *P* = 0.003, MUS vs. MUS + BIC: *P* = 0.266). (E) and (F) Effect of MUS (n = 9 cells from 5 wild type mice, Paired sample t test: Vehicle vs. MUS: t = 10.033, *P* < 0.0001, MUS vs. MUS + BIC: t = -16.764, *P* < 0.0001) or/and BIC (n = 8 cells from 5 wild type mice, Paired sample t test: Vehicle vs. MUS: t = -3.768, *P* = 0.007, MUS vs. MUS + BIC: t = 0.326, *P* = 0.754) on the firing frequency of VTA DA neurons from wild type mice in vivo. (G) and (H) Effects of MUS (n = 9 cells from 5 wild type mice, Wilcoxon's symbolic rank test: Vehicle vs. MUS: *P* = 0.008, MUS vs. MUS + BIC: *P* = 0.008) or/and BIC (n = 8 cells from 5 wild type mice, Paired sample t test: Vehicle vs. MUS: t = -10.466, *P* < 0.0001, MUS vs. MUS + BIC: t = -1.971, *P* = 0.089) on the percentage of spikes in bursts in vivo. Data are expressed as mean ± SEM; **P* < 0.05, ***P* < 0.01, ****P* < 0.001, *****P* < 0.0001, ns means no significant.


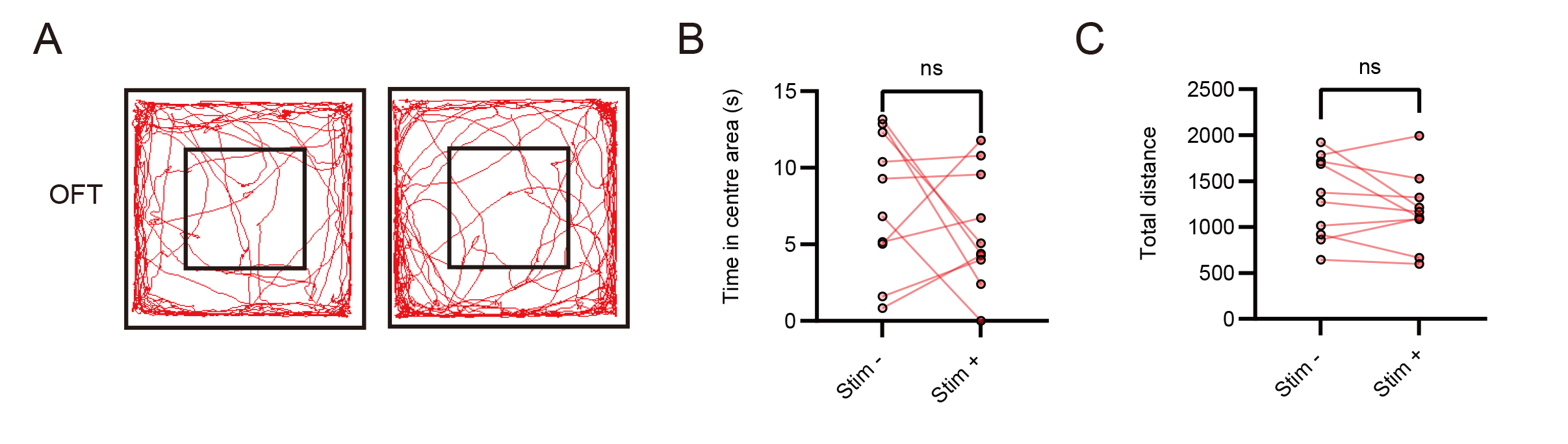


**Supplementary Fig. 4. Activation of GABA neurons in the RMTg region did not alter motor function in susceptible mice.** (A) Representative traces of susceptible mice during the open field test which were injected with AAV9-GAD-Cre and AAV-Retro-EF1a-DIO-hChR2-EYFP. (B) Time spent in the centre area of susceptible mice in open field (Paired sample t test, no stimulation vs. stimulation: t = 0.984, *P* = 0.351, n = 10 susceptible mice). (C) Total distance in open field of susceptible mice (Paired sample t test, no stimulation vs. stimulation: t = 1.469, *P* = 0.176, n = 10 susceptible mice). Data are expressed as mean ± SEM; **P* < 0.05, ***P* < 0.01, ****P* < 0.001, *****P* < 0.0001, ns means no significant.
